# Contract choice and advance selling strategy in a supply chain of FAP

**DOI:** 10.1371/journal.pone.0265661

**Published:** 2022-03-24

**Authors:** Jiaping Xie, Dan Liu, Ling Liang, Qinglin Li

**Affiliations:** 1 College of Business, Shanghai University of Finance and Economics, Shanghai, 200433, PR China; 2 Institute of Artificial Intelligence and Change Management, Shanghai University of International Business and Economics, Shanghai, PR China; Szechenyi Istvan University: Szechenyi Istvan Egyetem, HUNGARY

## Abstract

The advance selling (AS) has been widely applied in fresh industry for it can elevating the customer experience and increase flexibility thus profit for a retailer. However, the introduction of the AS will have an impact on spot market in pricing strategy, market share and the profit of the retailer. Hence, to coordinate the supply chain and improve the efficiency of the agricultural supply chain, a two-stage game theory model is constructed to analyze the effects of AS on three classic contracts: wholesale price, quantity discount and revenue-sharing contract. This paper also discusses the boundary conditions of whether a retailer should sell in advance. The conclusions of this paper are as follows: First, revenue-sharing contracts are superior to wholesale price and quantity discount contracts when retailers sell in advance, the wholesale price contract can perform better than the quantity discount contract in the presence of AS if the contract parameter is properly set. Second, a revenue-sharing contract that normally coordinates the supply chain can performs poorly when the retailer sells in advance that the social welfare would be higher if using a quantity discount contract instead. These conclusions have important implications for suppliers when retailers sell in advance. Such suppliers need to design appropriate contracts to distribute FAP that carefully take into consideration the AS activities in the market.

## 1. Introduction

As the social interaction tends to accelerate the spreading of the COVID-19 which broke out in Spring Festival 2020, the Chinese government has encouraged citizens to stay at home to slow the spread of the virus, which has caused a great loss to offline sales of fresh products. Against this circumstance, ordering fresh agricultural products (FAP) through e-commerce has gained popularity in China, aided by social platforms, taking the community as the unit, electronics retailers of FAP offering advance selling (AS) blowing across the country. According to Quest Mobile, the number of monthly active users on the WeChat mini program industry version reached 88.47 million in May 2020, increased by 65.6 percent compared with the previous month, and the channel proportion increased from 2% to 11.9% after the outbreak. There are two factors contributing to the rapid development of the e-commerce of FAP. The first is that consumers prefer shopping via the internet to avoid exposure to risky environments. The second is the fierce competition among e-commerce platforms in the field of fresh food, those fresh products could price even lower online than offline. The tough competition prompts e-commerce platforms to compete on price in the quest for market share, which has seriously affected the efficiency of the agricultural supply chain.

Different from general industrial products, FAP has the features of the perishable and short sales cycle, which will lead to a high risk of unsalable or out of stock in circulation process [1,2]. While AS not only can forecast the market demand and update the output in advance through the consumption information, but also enforce price discrimination and thus bring a higher profit for retailers. Therefore, AS has been widely applied in practice for it can effectively alleviate the uncertainty risk in the circulation process of FAP and improve the operational efficiency of the supply chain [3,4]. Most of fresh retailers such as Freshhema, Suning, Original Life and other large fresh retailers had already sold products in advance one after another. Chu Orange’s order volume exceeded 500 tons in half a month, accounting for 25%~30% of the total AS on the e-commerce platform Original Life in 2008, which become a successful example of AS. It adopts the mode of selling first and purchase later to reduce the loss caused by unsold goods in stores/front warehouses, which not only saves the circulation cost of logistics and storage but also alleviate the problem of large, packaged goods, short-term goods can not be sold in small stores.

Although AS and contract choice have been extensively studied in the literature, inefficient channel incentive will damage the efficiency of the agricultural supply chain in AS market has received very limited attention in the literature at present. According to the report of Guosen Securities Research Institute in 2020, FAP became such a staple in family life that become a way of e-commerce platform to obtain online traffic at a lower cost. Intense price competition not only disturbs the normal market price order, but also causes many adverse effects on suppliers. Therefore, effective channel management and contracts plays an important role in controlling AS market activities.

With the popularity of AS as an option to sell in advance in the fresh industry, a growing number of retailers observed this market strategy can profit for a retailer by elevating the customer experience and increasing the flexibility of the supply chain. Therefore, designing an appropriate choice of contracts is crucial for suppliers that distribute FAP when the retailer sells in advance. Our analysis tends to address the following questions: What are boundary conditions under which a retailer should sell in advance under different contracts? The introduction of the AS will undoubtedly have an impact on spot sales in pricing strategy, market share and retailer’s profit. To coordinate the supply chain and improve the efficiency of the entire chain, how does the supplier design contract?

To solve the above problems, this paper develops a two-stage game theory model to analyze the impacts of the AS on three classic contracts, wholesale price, quantity discount and revenue-sharing contract, and provide insights into retailer’s decision whether to sell early to strategic consumers. Moreover, the effect of the AS on social welfare under different contracts is also considered.

The results of our research indicate that in the wholesale price contract, if the supplier’s wholesale price is low, the retailer can gain more profits resulting from price discrimination through market segmentation, which is consistent with results by Zhao et al. Furthermore, we further consider the boundary conditions under which a retailer should sell in advance under quantity discount contracts and revenue sharing contracts, the results show that whether a retailer should to sell in advance are not only depends on the wholesale price, but also related to the freshness of the agricultural products and other factors.

This paper is organized as follows. First, we present a review of relevant literature in Section 2. Section 3 exhibits the structure and assumptions of our model and the benchmark scenario. We then examine the performance of the wholesale price, quantity discount and revenue sharing contract in Section 4 when the retailer sells early to strategic consumers. Section 5 analyzes social welfare posed in different contracts. Section 6 carries out a numerical simulation on the conclusion of this paper. Section 7 is the discussion and conclusion of this paper.

## 2. Literature review

We build upon three research distinct streams: advance selling, fresh agricultural supply chain contracting and supply chain coordination.

The literature related to AS focuses on the question of whether a retailer should sell in advance, Cho and Tang [5] examine the impact of AS for seasonal products on the performance of the supply chain, showing that combined advance and regular selling is more advantageous for suppliers and AS only is more beneficial for retailers. Zhao et al. [6] show that whether retailer’s AS option is beneficial to suppliers or retailers depends on the marginal cost of products. Cachon [7] argues that a pre-sale discount contract that coordinates the supply chain which makes the retailer take the inventory risk of pre-season order, while the supplier bears the production risk with exceeding quantity of order. Gilbert and Ballou [8] uncover that forwarding order contracts under certain conditions offered by dealers can reduce the cost of each member in the steel supply chain. Cvsa and Gilbert [9] show that suppliers offering pre-orders for short life cycle products can benefit both suppliers and buyers when compared to no pre-orders. Some scholars combined AS and financing, Gupta and Chutani [10] examine the financing problem in the case of supply interruption, which concluded that presale can alleviate the impact of supply interruption, at the same time it can increase the profit of the overall supply chain. Jin et al. [11] compared the impact of two financing strategies of pre-sale and delayed payments on retailer’s performance, which indicate that the pre-sale is more suitable for the retailer when retailers have sufficient capital or customers are relatively sensitive to prices, while the delayed payment is preferable for both the retailer and the supply chain when the retailer’s capital is limited. Niu and Yang [12] analyze the influence of presale on the three-level supply chain under the social influence, which found that the double advance sales discount model can realize Pareto improvement of supply chain although it cannot coordinate the supply chain when compared with no AS. Wei and Zhang [13] show that the production decision that relies on the pre-sale goal can effectively reduce the strategic waiting behavior of consumers and thus improve the profit of sellers. Zhang et al. [14] found that AS is always beneficial to suppliers but can harm the profits of retailers, it can achieve Pareto improvement under certain conditions and thus is favorable to the supply chain. Peng et al. [15] studied the influence of two different price guarantee policies on retailer’s profit. Xiao et al. [16] investigate the equilibrium strategies under dynamic pricing scheme and price commitment scheme when a seller sells in advance. Zhang et al. [17] discusses the effect of partial refund strategy on retailers’ profit in the service industry environment where demand and consumption uncertainty are the main influencing factors. In addition, there are some scholars studies the pricing policy based on Newsvendor model [18,19]. The literature above indicates that the AS can increase the profit of the supply chain and alleviate the budget constraint, but the research mentioned above mainly concentrates on the steel industry, seasonal products, short product life cycle and other general products, the issue of FAP is not considered within those studies which are the focuses of this paper.

Some papers consider the problem of agricultural supply chains pricing in the presence of strategic consumers. Shao and Lu [20] develop a F2F model for the pricing of FAP. Tang et al. [21] research the pricing strategy under the combination of “agriculture-supermarket jointing” and e-commerce channels of FAP considering quality loss. Wang and Li [22] propose a pricing method for dynamically identifying the shelf life of food to reduce the loss of FAP. Chew et al. [23] adopt the stochastic dynamic programming method to study the order quantity and pricing of perishable products with multi-cycle life. Blackburn and Scudder [24] take the marginal value time of products as the measurement standard to minimize the lost value over time in the supply chain, which is conducted with the specific FAP. Tang et al. [25] analyze the pricing and inventory of FAP considering the strategic behavior of consumers based on the newsboy model. Dan and Ding [26] examine the second-replenishment policy for FAP under customer classification and results show that the policy can effectively reduce order volumes and benefit retailers. Wang and Dan [27] analyze a multi-variety ordering model for FAP with time-varying customer utility affected by freshness. Wan et al. [28] analyzes the conditions for coordinate the FAP supply chain under different supply chain structures. To reduce the loss of freshness, the above literature has studied the pricing and procurement in the supply chain of FAP.

Designing contracts to coordinate the supply chain of FAP had been getting a great deal of research. Yan et al. [29] quantify consumers’ strategic behavior into risk aversion coefficient and designed two kinds of FAP supply chain coordination contracts based on revenue sharing and wholesale price. Yan et al. [30] apply the Internet of Things (IoT) to the fresh produce (FAP) supply chain and coordinate the supply chain by revenue-sharing contract. Xiao et al. [31] establish a cost-sharing mechanism to coordinate the fresh supply chain of the CIF (Cost, insurance and freight) business model with long-distance transportation. Mohammadi et al. [32] design a new coordination contract named RPTIS, which significantly improved the freshness and survival rate of fresh products. Yan et al. [33] analyze the impacts of choosing different transportation modes of the supply chain under revenue sharing and cost-sharing contracts. Moon et al. [34] show that incremental quantity discount contracts can propel suppliers and retailers to jointly invest to maintain product freshness when taking into account the fairness, and thus realized supply chain coordination. Zhao et al. [35] examine the coordination of FAP in the dual-channel supply chain under the AS mode. Yang et al. [36] uncover the coordination of agricultural product supply chain contract with asymmetry of freshness in the upstream and downstream. Besides, some scholars analyze the coordination of agricultural product supply chain contracts when producers supply products to a distant market through third-party logistics (3PL) suppliers while distributors take efforts to maintain the freshness [37,38].

The aforementioned literature studies contract coordination in a fresh agricultural supply chain, but in real practice, some contracts are costly and still have some wrinkles to be ironed out practice. This paper studies several classical supply chain contracts and considers the optimal pricing in the fresh agricultural supply chain. Besides, if the retailer sells in advance, he may blindly reduce the market price in AS period for market share, which affects the efficiency of the supply chain and causes many adverse effects on suppliers. To alleviate the AS impact on spot sells in pricing strategy, market share and retailer’s profit, how the supplier design appropriate contracts to improve the efficiency of the supply chain is the emphasis of our study.

The most relevant research to our paper is Zhao et al. [6], who mainly examines the impacts of AS in wholesale price contract. In this study, we integrate the concept of freshness into the model, which is the main characteristic of agricultural products. Analyzing the impacts of AS on three classic contracts: wholesale price, quantity discount and revenue sharing contract, and discuss the boundary conditions of the retailer whether or not to sell early to strategic consumers in each contract. Since we analyze the effects of AS on three different contracts, our results are different from Zhao et al.

## 3. Mode description and the benchmark

### 3.1 Mode description

Consider the supply chain with one supplier(she) selling a single fresh agricultural product through a retailer(he). We focused our analysis on three contracts: the wholesale price contract, the quantity discount contract and the revenue sharing contract. The wholesale price contract is easy to enforce and is considered a benchmark in the literature, although it generally does not coordinate the supply chain. If contract parameters are properly selected, quantity discount contract and revenue sharing contract can coordinate supply chain, while other contracts are more relevant to coordinate supply chain under uncertain demand.

**Fig 1:**
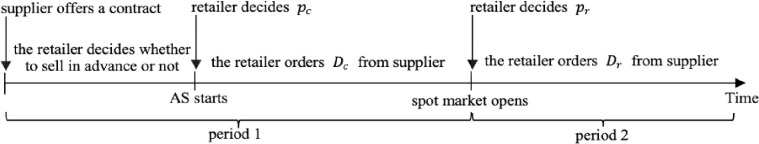
Sequence of events.

In practice, the supplier signs a contract with the retailer before the AS period starts, but the retailer can make the decisions after the AS period begins. Therefore, we consider a Stackelberg game with the supplier as the dominant. Our model has two periods, a schematic of the sequence of events for each period as shown in Fig 1, and the game proceeds as follows:

Stage 1: The supplier designs a contract for the retailer to the AS period, and the retailer needs to decide whether to sell in advance or not.

Stage 2: In period 1, if the retailer sells in advance, then the retailer will provide the product with a pre-order price *p*_*c*_ to consumers and orders *D*_*c*_ units from the supplier.

Stage 3: In period 2, the retailer purchase *D*_*r*_ units from the supplier and sells the product at a spot price *p*_*r*_ to consumers.

The freshness of the FAP decay with the elapse of time, for the FAP belongs to seasonal and shorter shelf-life items. To provide a precise description of the effect of time variation of agricultural products’ freshness on consumer utility, a freshness function i.e., *l* = *e*^−*βt*^ described that the freshness is decreased with the elapse of time, where *β*>0 is the freshness decay index, which represents the rate at which freshness decline with time, *t*∈(0,1) represents the time when the consumer receives the product on spot market. At the end of the AS period, consumers can get the pre-order product, when *t*→0, the freshness of agricultural products is 1, which means the consumer who buys the product in AS period is assumed to be the highest freshness degree of agricultural products, when *t*→1, the freshness of agricultural products is *e*^−*β*^.

We assume the size of the market is standardized to 1, parameter *θ*∈[0,1] denotes the consumer’s valuation of the product, its probability density function is *g*(*θ*), which obeys the uniform distribution. Consumers will choose to buy products between the AS period and spot market by comparing the utility surplus. The utility of a consumer choosing to buy fresh goods in AS period is expressed as *u*_*c*_ = *θ*−*p*_*c*_, while the utility in spot market is *u*_*r*_ = *e*^−*βt*^*θ*−*p*_*r*_.

In a model of two-stage game theory, the second stage spot market is always the second-best choice for consumers. Therefore, a consumer with the valuation of *θ* will choose to buy in AS period under the condition of *u*_*c*_≥*u*_*r*_ and *u*_*c*_≥0. Similarly, a consumer will buy products in spot market if *u*_*r*_≥*u*_*c*_ and *u*_*r*_≥0. Since *u*_*c*_−*u*_*r*_ is a monotonically increasing function to *θ*, there exists a *θ*∈(0,1) such that *u*_*c*_ = *u*_*r*_. The parameter *θ* would then segment the consumer into AS and spot market demand, then the fraction of the consumer that corresponds to AS period is $D_{c}=1-\frac{p_{c}-p_{r}}{1-e^{-\beta t}}$, and the fraction of the consumer that corresponds to spot market is $D_{r}=\frac{p_{c}-p_{r}}{1-e^{-\beta t}}-\frac{p_{r}}{e^{-\beta t}}$.

Table 1 summarizes the basic symbols used throughout the paper. We denote *c* to express the optimal decision for the centralized supply chain, *w* to represent the optimal decision for the wholesale price contract, *QD* to represent the optimal decision for the quantity discount contract, *RS* to represent the optimal decision for the revenue-sharing contract. We use subscripts *s*, *r* and *SC* to refer to suppliers, retailers, and supply chains. In addition, we add *N* in the superscript to indicate the situation that the retailer does not sell early to strategic consumers.

**Table 1:** Decision variables and parameters.

*c*	procurement cost of supplier
*θ*	consumer’s valuation of fresh product
*w*	supplier’s wholesale price
*b*	slope of the classic quantity discount contract
*λ*	retailer’s percentage of the returns under the revenue sharing contract
*β*	the freshness decay index, which represents the speed of freshness decay with time
*t*	the time when the consumer receives the product in spot market
*D*_*c*_,*D*_*r*_	the fraction of the consumer that corresponds to AS period, spot market
*p*_*c*_, *p*_*r*_	pre-order price, spot price
*π*_*r*_,*π*_*s*_, *π*_*sc*_	profit of the retailer, supplier, and supply chain
*SW*, *CS*	social welfare, consumer welfare

### 3.2 The benchmark

As one benchmark scenario, we first analyze the case when retailers do not sell in advance. In this situation, consumers who wish to obtain the product can only buy from spot market. If the supplier and the retailer are integrated, the profit of the supply chain will be $\left(p_{r}-c)(1-\frac{p_{r}}{e^{-\beta t}}\right)$, define *a* = *e*^−*βt*^, then the optimal retail price is $p_{r}^{c^{N}}=\frac{a+c}{2}$, the optimal profit is $\pi_{sc}^{c^{N}}=\frac{{\left(a-c\right)}^{2}}{4a}$.

As a Stackelberg leader of the supply chain, the supplier first determines the wholesale price, then the retailer determines the retail price. The profit of the retailer will be $\left(p_{r}-w^{N})(1-\frac{p_{r}}{a}\right)$, and the profit of the supplier is $\left(w^{N}-c)(1-\frac{p_{r}}{a}\right)$. We use backward induction to solve this problem, and the supplier’s optimal wholesale price and the retailer’s optimal spot price $p_{r}^{N}=\frac{a+w^{N}}{2}$, If the retailer not to sell early, the wholesale price contract fails to coordinate the supply chain unless *w*^*N*^ = *c* and the supplier earn zero profit. The optimal retail, supplier and supply chain profit can be expressed as:

$$\pi_{r}^{w^{N}}=\frac{{\left(a-w^{N}\right)}^{2}}{4a},\;\pi_{s}^{w^{N}}=\frac{\left(w^{N}-c)(a-w^{N}\right)}{2a},\;\pi_{SC}^{w^{N}}=\frac{\left(a-w^{N})(a+w^{N}-2c\right)}{4a}$$ (1)

Suppose the supplier offers the retailer an all-unit quantity discount contract in the quantity discount contract. The retailer has to pay the supplier *w*(*q*_*r*_^*N*^) = *k*−*bq*_*r*_^*N*^ per unit when the order quantity is *q*_*r*_^*N*^, thus the retailer’s profit will be $[p_{r}-w({q_{r}}^{N})](1-\frac{p_{r}}{a})=b-k+\frac{(a-2b+k)p_{r}}{a}-\frac{(a-b){p_{r}}^{2}}{a^{2}}$. The optimal spot price satisfies the first-order condition is $p_{r}^{QD^{N}}=\frac{a(a-2b+k)}{2(a-b)}$. If $k=c+b(1-\frac{c}{a})$, then $p_{r}^{QD^{N}}=p_{r}^{c^{N}}$ and the quantity discount contract can coordinate the supply chain. The optimal retail, supplier and supply chain profit expression obtained as:

$$\pi_{r}^{QD^{N}}=\frac{(a-b){\left(a-c\right)}^{2}}{4a^{2}},\;\pi_{s}^{QD^{N}}=\frac{b{\left(a-c\right)}^{2}}{4a^{2}},\;\pi_{SC}^{QD^{N}}=\frac{{\left(a-c\right)}^{2}}{4a}$$ (2)

Under a revenue-sharing contract, the supplier sets a relatively low wholesale price $w^{{RS}^{N}}$ and the retailer share 1−*λ* percentage of the revenues returns to the supplier. The supplier decides the wholesale price $w^{{RS}^{N}}$ and the revenue-sharing ratio *λ*(0≤*λ*≤1). The retailer’s profit will be $\lambda p_{r}(1-\frac{p_{r}}{a})-w^{{RS}^{N}}(1-\frac{p_{r}}{a})$, then the optimal retail price $p_{r}^{RS^{N}}=\frac{w^{{RS}^{N}}+a\lambda }{2\lambda }$, if $w^{{RS}^{N}}=c\lambda$, then $p_{r}^{RS^{N}}=p_{r}^{c^{N}}$, and the revenue-sharing contract can coordinate the supply chain. The optimal retail, supplier and supply chain profit are the following:

$$\pi_{r}^{RS^{N}}=\frac{\lambda {\left(a-c\right)}^{2}}{4a},\;\pi_{s}^{RS^{N}}=\frac{(1-\lambda ){\left(a-c\right)}^{2}}{4a},\;\pi_{SC}^{RS^{N}}=\frac{{\left(a-c\right)}^{2}}{4a}$$ (3)

## 4. Supply chain with AS

### 4.1. Centralized supply chain

Taking the AS into consideration, if the supplier and the retailer are integrated, in period 2, the supply chain determines spot price $p_{r}^{c}$ to maximize its profit. In period 1, the centralized supply chain seeks profit maximization by deciding $p_{c}^{c}$, the centralized supply chain’s optimization problem can be formally formulated as a two-stage maximization problem:

$$\underset{p_{c}^{c}}{max}\left\{p_{c}^{c}D_{c}-c(D_{c}+D_{r})+\underset{p_{r}^{c}}{max}\{p_{r}^{c}D_{r}\}\right\}$$ (4)

Solving the two-stage maximization problem in (4), we have proposition 1. The proof of proposition 1 and other proofs of propositions and corollaries are included in the S1 Appendix.

**Proposition 1.** If $0<a<\frac{2}{3}$ and 0<*c*<*c*_1_, then the centralized supply chain will sell in advance and the optimal spot price $p_{r}^{c}=\frac{a(1-a)(2+c)}{2(4-3a)}$, the optimal pre-order price $p_{c}^{c}=\frac{\left(1-a)(2+c\right)}{4-3a}$, the optimal centralized supply chain’s profit $\pi_{SC}^{c}=\frac{4-12c+c^{2}-a(4-8c+c^{2})}{16-12a}$. Otherwise, the centralized supply chain will not sell in advance and sets $p_{r}^{c^{N}}=\frac{a+c}{2}$. In addition, $p_{r}^{c}<p_{r}^{c^{N}}$.

If the retailer sells in advance, it impacts the supply chain both positively and negatively. On the one hand, it indirectly helps the retailer conduct market segmentation, which can enforce price discrimination and thus profit for a retailer. On the other hand, AS induces some consumers who originally buy goods from spot market to purchase ahead in AS period, thereby cannibalizing supply chain demand in spot market. Proposition 1 indicates that the first effect will outweigh the second and the centralized supply chain will benefit from the AS if the procurement cost of fresh suppliers is low, and the freshness loss of FAP is small in spot market. The reason is as follows: first, the procurement cost of the fresh supplier must be kept within a low range, otherwise, high procurement cost will make retailers impossible to provide lower spot prices and get positive profits in spot market. Secondly, when the freshness decay index is high or consumers take longer to get the spot products, then consumers are more likely to buy in advance, thus AS becomes a strong competitor. In this situation, the supply chain should sell in advance.

When the centralized supply chain sells in advance, the spot price will be lower than with no AS. For the AS indirectly segment the market by offering the product at a higher price to customers who with high valuations and more willing to buy products during the AS period, by offers relatively lower prices to consumers who with low valuations and willing to buy in spot market.

### 4.2. Wholesale price contract

Suppose the supplier charges the retailer a wholesale price *w*. In period 2, the retailer determines his optimal price $p_{r}^{w}$. In period 1, the retailer maximizes his profit by determining $p_{c}^{w}$. The retailer’s optimization problem can be formally written as a two-stage maximization problem:

$$\underset{p_{c}^{w}}{max}\left\{p_{c}^{w}D_{c}-w(D_{c}+D_{r})+\underset{p_{r}^{w}}{max}\{p_{r}^{w}D_{r}\}\right\}$$ (5)

Solving the optimization problem in (5), we get proposition 2.

**Proposition 2.** If $0<a<\frac{2}{3}$ and $w<\tilde{w}$, then the retailer will sell in advance and the optimal spot price $p_{r}^{w}=\frac{a(1-a)(2+w)}{2(4-3a)}$, the optimal pre-order price $p_{c}^{w}=\frac{\left(1-a)(2+w\right)}{4-3a}$, the optimal profits of the retailer $\pi_{r}^{w}=\frac{4-12w+w^{2}-a(4-8w+w^{2})}{4(4-3a)}$, the optimal profits of the supplier $\pi_{s}^{w}=\frac{\left(w-c)[6-a(4-w)-w\right]}{2(4-3a)}$. Otherwise, the retailer will not sell in advance and sets $p_{r}^{N}=\frac{a+w}{2}$. When the retailer sells in advance, the wholesale price contract fails to coordinate unless *w* = *c* and the supplier earn zero profit.

Proposition 2 indicates that the supplier’s wholesale price and is the freshness loss of FAP in spot market is the key factor affecting whether the retailer sells in advance, which is similar to the results of centralized supply chain. If the supplier’s wholesale price is low, the retailer can gain more profits resulting from price discrimination through market segmentation. Otherwise, double marginalization prevents retailers sells early to strategic consumers.

**Corollary 1.** The effects of sells in advance in the wholesale price contract are as follows:

1. $p_{r}^{w}<p_{r}^{w^{N}}$;
2. If $a<\frac{1}{6}(2-w^{2}+\sqrt{4+20w^{2}+w^{4}})$ and $c_{2}<c<1,\;\pi_{SC}^{w}<\pi_{SC}^{w^{N}}$.

Corollary 1 shows that the double marginalization lowered the spot price due to the retailer sells in advance. Besides, the AS impacts the supply chain both in two ways. On the one hand, it expanded the total market demand. On the other hand, it cannibalized supply chain demand in spot market and lowered the profit margin of the retailer, which result in lower profit. In the scenario involving high procurement cost and the low freshness of agricultural products in spot market, the second effect will be outweighing the first and the supply chain will be harmed by AS.

**Corollary 2.** The pre-order price and profit of retailer decreases with *a*; The profit of supplier increases with *a*.

Corollary 2 represents when the freshness of the products is relatively low in spot period, the pre-order price decreases with *a*. The total consumer demand $1-\frac{p_{r}}{a}$ increases with *a*, and the profit of the supplier increases with the total consumer demand. which ultimately led to the profit of the supplier increases with *a*.

### 4.3 Quantity discount contract

Under the quantity discount contract, the retailer has to pay the supplier *w*(*q*_*r*_) = *b*(1−*c*)+*c*−*bq*_*r*_, where *b*(0<*b*<1) is the quantity discount contract parameter. In period 2, the retailer determines his optimal price $p_{r}^{QD}$. In period 1, the retailer maximizes his profit by deciding $p_{c}^{QD}$. The retailer’s optimization problem can be formally written as a two-stage maximization problem:

$$\underset{p_{c}^{QD}}{max}\left\{p_{c}^{QD}D_{c}-w(q_{r})(D_{c}+D_{r})+\underset{p_{r}^{QD}}{max\{}p_{r}^{QD}D_{r}\}\right\}$$ (6)

Solving the optimization problem in (6), we get proposition 3.

**Proposition 3.** If *b*_2_<*b*<1, then the retailer will sell in advance and the optimal spot price $p_{r}^{QD}=\frac{a(1-a)[2+c-b(1-c)]}{2[4-b-a(3-b)]}$, the optimal pre-order price $p_{c}^{QD}=\frac{\left(1-a)[2+c-b(1-c)\right]}{\left[4-b-a(3-b)\right]}$, the optimal profits of the retailer $\pi_{r}^{w}=\frac{4-12w+w^{2}-a(4-8w+w^{2})}{4(4-3a)}$, Otherwise, the retailer will not sell in advance and sets $p_{r}^{QD^{N}}=\frac{a(a-2b+k)}{2(a-b)}$. When the retailer sells in advance, the quantity discount contract fails to coordinate unless *w*(*q*_*r*_) = *c* and then $p_{r}^{QD}=p_{r}^{c}$.

Proposition 3 reveals that under a traditional quantity discount contract, the retailer will sell in advance when the discount is large enough. Meanwhile, Proposition 3 states that quantity discount contracts are very limited in coordinating supply chains when AS and spot market coexists. Despite the quantity discount contract can induce the retailer to reduce the spot price by increasing the amount of discount, and then ultimately increase the total demand of the market, it will also induce more demand in AS period and eventually make the total demand of the consumer exceed the optimal level of supply. In addition, the supplier needs to coordinate two decisions of the retailers: the AS quantity and spot market quantity. If the payment plan depends only on the order quantity, the supplier will not be able to coordinate the ordering decision between the two channels. Therefore, the quantity discount contract cannot coordinate the supply chain when AS and spot markets coexist.

**Corollary 3.** The effects of sells in advance in the quantity discount contract are as follows:

1. $p_{r}^{QD}<p_{r}^{c}$.
2. $p_{r}^{QD}<p_{r}^{QD^{N}}$.
3. $\pi_{s}^{QD}<\pi_{s}^{QD^{N}}$.

Corollary 3 indicates that the spot price in quantity discount contract is lower than that in a centralized supply chain when retailer sells advance, the spot price gets lower if retailer sells advance for the double marginalization lowered the spot price. The supplier is always worse off with AS, for the quantity discount contract can coordinate the supply chain when only regular selling exists but fails to coordinate the supply chain when combined advance and regular selling. The effects of the AS on a retailer’s profit are uncertain and depend on whether the benefit from the AS outweighs the profit loss to spot market.

### 4.4 Revenue sharing contract

The supplier offers the retailer a revenue-sharing contract (*w*^*RS*^, *λ*), where *w*^*RS*^ represents wholesale price and *λ* is the revenue-sharing ratio, in period 2, the retailer determines his optimal price $p_{r}^{RS}$. In period 1, the retailer maximizes his profit by deciding $p_{c}^{RS}$. The retailer’s optimization problem can be formally written as a two-stage maximization problem:

$$\underset{p_{c}^{RS}}{max}\left\{\lambda p_{c}^{RS}D_{c}-w^{RS}(D_{c}+D_{r})+\lambda \underset{p_{r}^{RS}}{max}\{p_{r}^{RS}D_{r}\}\right\}$$ (7)

Solving the optimization problem in (7), we get proposition 4.

**Proposition 4.** The revenue-sharing contract can coordinate the supply chain if *w*^*RS*^ = *cλ*, and the coordinating RS contract results in the optimal profit of supplier, retailer and the supply chain is:

$$\pi_{r}^{RS}=\lambda \pi_{SC}^{c},\;\pi_{s}^{RS}=(1-\lambda )\pi_{SC}^{c},\;\pi_{SC}^{RS}=\pi_{SC}^{c}$$ (8)

Since the RS contract can coordinate the supply chain, then the boundary conditions under which a retailer should sell in advance is the same as the 4.1 centralized supply chain scenario. The revenue-sharing contract is superior to wholesale price contract and quantity discount contract for it can perfectly coordinate the supply chain if combined advance and regular selling.

The following is a comparison between the wholesale price contract and the quantity discount contract and examines which contract can better serve the supply chain with AS.

**Proposition 5.** There exists a wholesale price *w*_2_>*c*, such that $\pi_{SC}^{w}>\pi_{SC}^{QD}$ when *c*<*w*<*w*_2_.

Proposition 5 shows that the wholesale price contract is superior to the quantity discount contract if the contract parameter is properly set, despite the wholesale price contract usually failing to coordinate the supply chain for double marginal. In our model, the AS poses competition with spot market channel, so the double marginal effect of the wholesale price contract is reduced when the retailer sells in advance.

The proposition 5 have important implications for suppliers when the retailer sells in advance. Such suppliers need to choose appropriate contracts to distribute FAP that carefully take into consideration the AS activities in the market. If she sets a lower wholesale price in the wholesale price contract, the quantity discount contract is more beneficial to total supply chain’s profit, otherwise the wholesale price contract is more profitable.

## 5. Social welfare

In this section, we examine the implications of the AS on social welfare. From the perspective of social welfare, the AS can be beneficial for the AS to pose competition with spot market channel, which can lead to lower prices and provide more purchase choices for consumers. Moreover, if the retailer sells in advance, then the retailer can enforce price discrimination and thus profit for the supply chain.

For the centralized supply chain, if the retailer does not sell in advance, the consumer welfare and social welfare:

$$CS^{c^{N}}=\int_{\frac{1+c}{2}}^{1}\left(x-\frac{1+c}{2}\right)dx=\frac{{\left(1-c\right)}^{2}}{8}$$ (9)

$$SW^{C^{N}}=\pi_{sc}^{c^{N}}+CS^{c^{N}}=\frac{2(a^{2}+c^{2})-a[c(6-c)-1]}{8a}$$ (10)

For the quantity discount contract and the revenue-sharing contract can coordinate the supply chain if the retailer does not sell in advance, then the consumer welfare and social welfare are the same as the centralized supply chain.

Under the wholesale price contract, if the retailer does not sell in advance, then

$$CS^{w^{N}}=\int_{\frac{a+w}{2}}^{1}\left(x-\frac{a+w}{2}\right)dx=\frac{{\left(a+w-2\right)}^{2}}{8}$$ (11)

$$SW^{w^{N}}=\frac{a^{3}+a[{\left(2-w\right)}^{2}-4c]+2a^{2}(w-1)+2w(2c-w)}{8a}$$ (12)

For the centralized supply chain, if the retailer sells in advance, thus

$${CS}^{C}=\int_{\theta }^{1}(x-p_{c})dx+\int_{\frac{p_{r}}{a}}^{\theta }(ax-p_{r})dx=\frac{4-c(1-a)(4-c)}{8(4-3a)}$$ (13)

$$SW^{C}=\frac{12-28c+3c^{2}-a(8-20c+3c^{2})}{8(4-3a)}$$ (14)

For the revenue-sharing contract can coordinate the supply chain if the retailer sells in advance, then the consumer welfare and social welfare are the same as the concentrated case.

Under the wholesale price contract, if the retailer sells in advance, then consumer welfare and social welfare:

$${CS}^{w}=\frac{4-w(1-a)(4-w)}{8(4-3a)}$$ (15)

$$SW^{w}=\frac{12-4c(6-w)-w(4+w)-a[8-4c(4-w)-w(4+w)]}{8(4-3a)}$$ (16)

Under the quantity discount contract, if the retailer sells in advance, then

$${CS}^{QD}=\frac{\left(\begin{array}{c} 4{\left[2-(1-b)c\right]}^{2}+a^{2}[2b(2+7c-3c^{2})-3c(4-c)+(3c^{2}-2c-1)b^{2}]+\\ a[28c-12-7c^{2}+(1+2c-7c^{2})b^{2}+2b(7c^{2}-15c-2)] \end{array}\right)}{8{\left[4-a(3-b)-b\right]}^{2}}$$ (17)

$${SW}^{QD}=\frac{\left(\begin{array}{c} 4[\begin{array}{c} 12-28c-3b^{2}c+3c^{2}\\ -b(4-16c+3c^{2}) \end{array}]+a^{2}[\begin{array}{c} 24+9c^{2}+b^{2}(1-10c+c^{2})\\ -2b(6-23c+5c^{2})-60c \end{array}]-\\ a[164c-68-21c^{2}-b^{2}(1-22c+c^{2})+2b(14-55c+11c^{2})] \end{array}\right)}{8{\left[4-a(3-b)-b\right]}^{2}}$$ (18)

**Proposition 6.** The comparison of consumer welfare and social welfare between the centralized supply chain with AS and without AS:

1. $CS^{C^{N}}<CS^{C}$;
2. If 0<*c*<*c*_4_, $SW_{C}^{N}<SW_{C}$; If *c*_4_<*c*<1, $SW_{C}^{N}>SW_{C}$.

Proposition 6 states that the introduction of the AS is beneficial from the consumers’ viewpoint, for it creates competition in the channel of spot market, thereby reducing spot price and providing more purchase choices for consumers. According to Proposition 1, when the procurement cost is low, the centralized supply chain will sell in advance, so the AS will benefit both consumers and the centralized supply chain. With the procurement cost gradually increasing, the negative effect of AS on the profits of the centralized supply chain is overweight the positive effect on consumer welfare, which ultimately leads to AS can cause damage to social welfare.

**Proposition 7.** If *w*>*c*, *SW*^*w*^<*SW*^*RS*^<*SW*^*QD*^.

The proposition means that although the revenue-sharing contract can perfectly coordinate the supply chain when retailer sells in advance, it does not always result in highest social welfare of the three contracts. The social welfare under revenue-sharing contracts is greater than wholesale price contracts but less than quantity discount contracts if the wholesale price is greater than procurement cost.

According to proposition 5 and 7, we can get that quantity discount contracts can bring more consumer welfare despite it cannot coordinate the supply chain from the perspective of consumers. Moreover, it can bring more social welfare when compared with wholesale price contracts.

## 6. Numerical analysis

In this section we investigate the functions of the optimal profit and social welfare under different contracts, the numerical examples use the following parameter values: *a* = 0.1, *c* = 0.3, *b* = 0.2.

**Fig 2:**
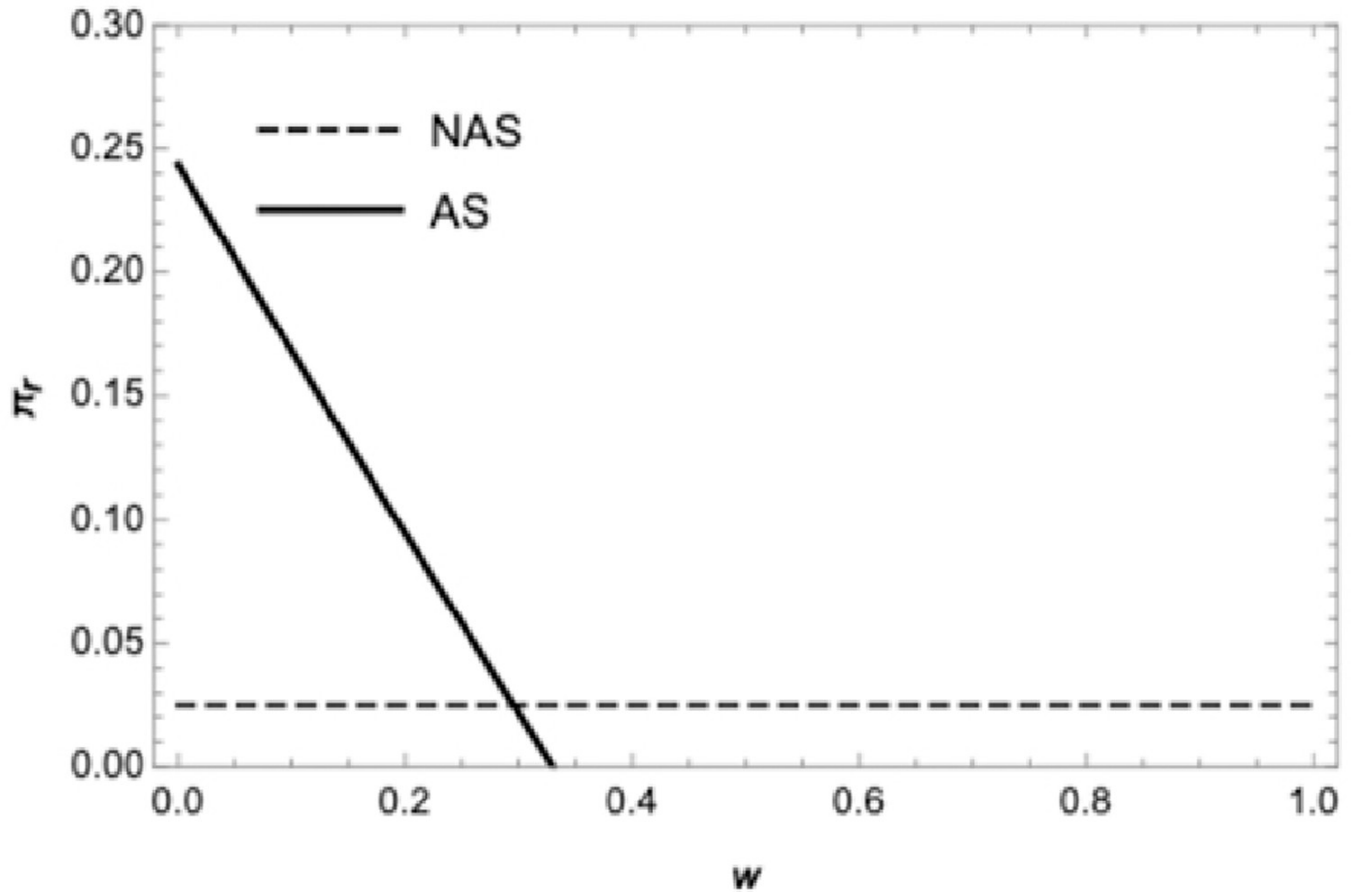
The retailer’s profit in the wholesale price contract.

**Fig 3:**
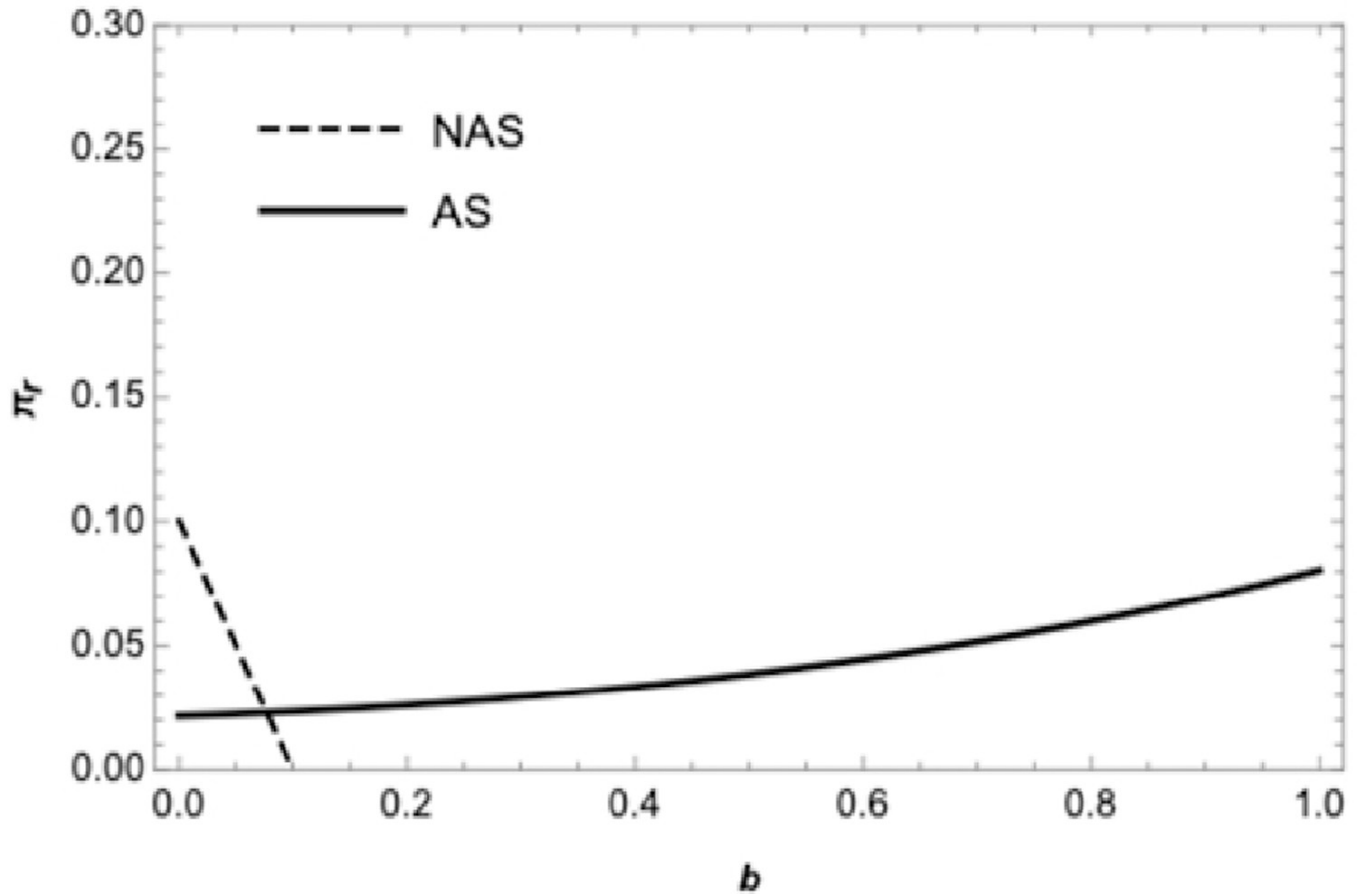
The retailer’s profit in the quantity discount contract.

**Fig 4:**
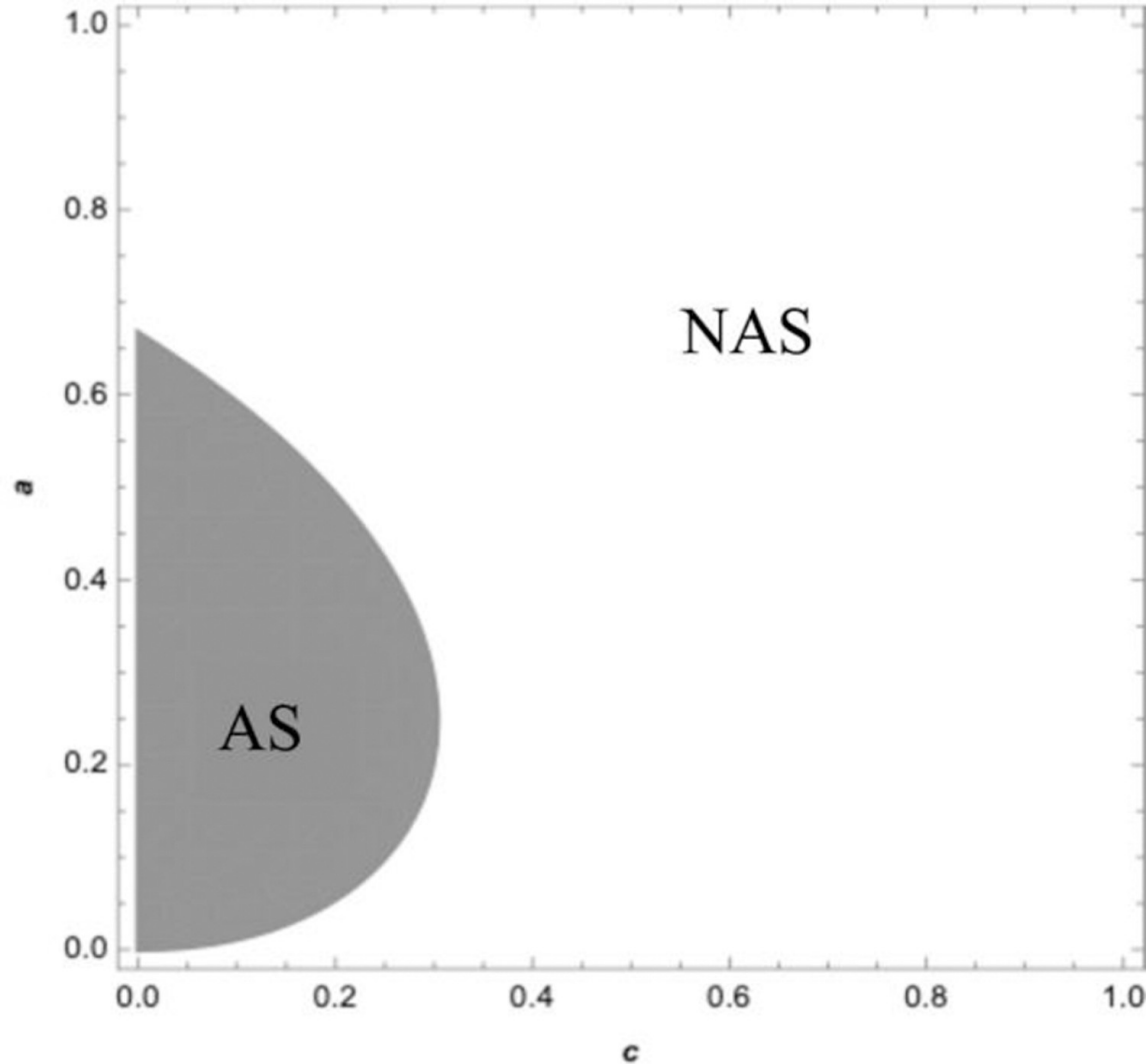
The regions for retailer sell in advance under the RS contract/centralized supply chain.

**Fig 5:**
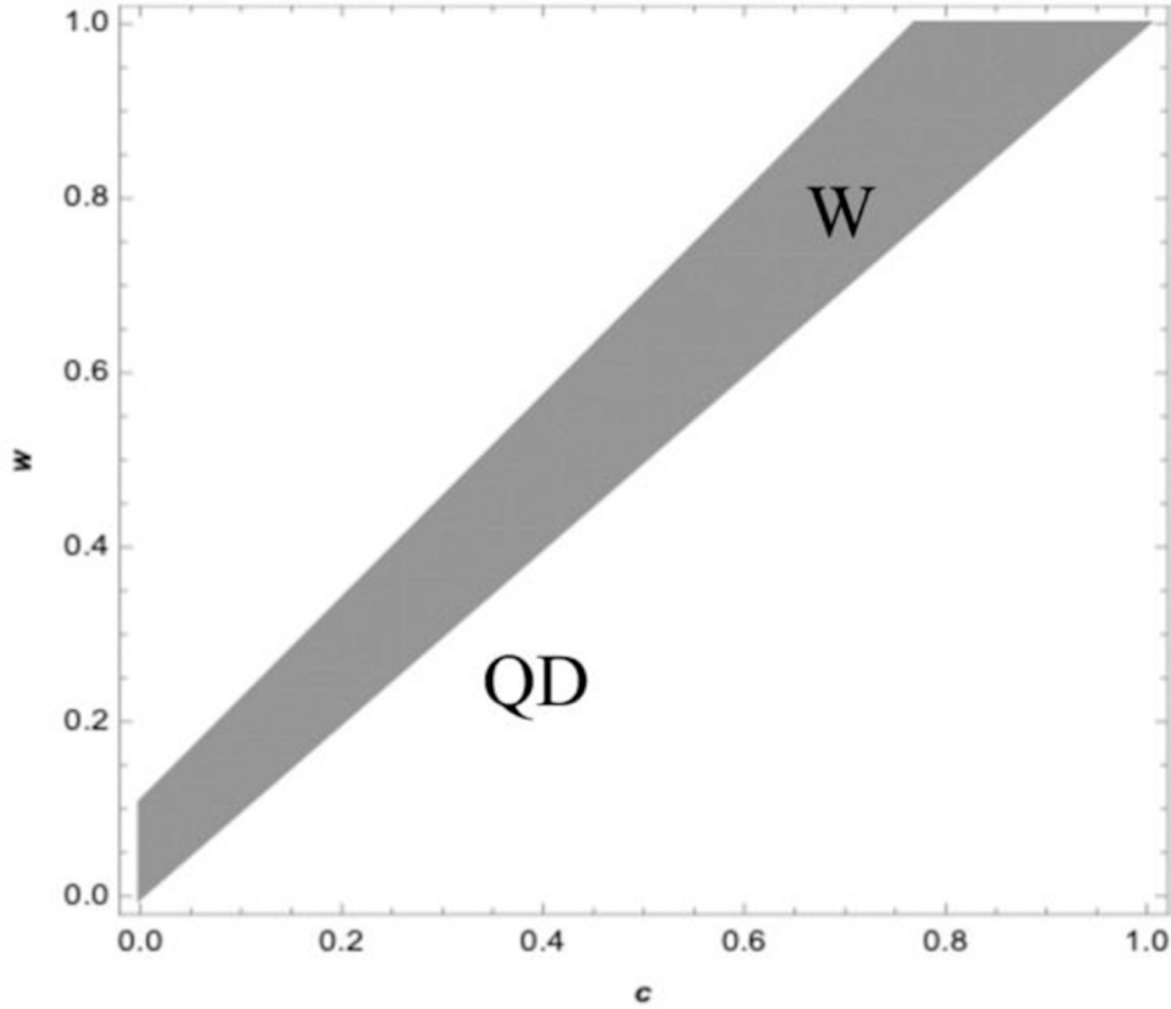
Optimal contract for supply chain.

Fig 2 illustrates the retailer’s profit curve under the wholesale price contract. The solid curve represents the retailer’s optimal profit if the retailer sells in advance, and the dashed curve represents the retailer’s optimal profit if the retailer does not sell in advance. In the wholesale price contract, when the supplier’s wholesale price is relatively low, the retailer can be better off by selling advance, with the increase of wholesale price, the retailer can be worse off by selling advance, this is consistent with the conclusions of proposition 2. Fig 3 displays the retailer’s optimal profit under the quantity discount contract. The solid curve represents the retailer’s optimal profit if the retailer sells in advance, and the dashed represents the optimal profit if the retailer does not sell in advance. Under the traditional quantity discount contract when the quantity discount is relatively large, it is a benefit for the retailer to sell in advance, which is consistent with the conclusion of proposition 3. Fig 4 exhibits the boundary conditions of a retailer should sell in advance under the revenue sharing contract. Graph corresponding to the shaded area shows the retailer can be better off by selling in advance under the revenue-sharing contract. It means that when the procurement cost and the freshness loss in the spot stage are both low, retailers can benefit from AS under the revenue sharing contract, which is consistent with the conclusion of proposition 4. Fig 5 corresponding to the shaded area shows the retailer can be better off by using the wholesale price contract compared with the quantity discount contract if the retailer sells in advance. There exists a wholesale price above the cost price that makes the wholesale price contract is superior to the quantity discount contract, which is consistent with the conclusion of proposition 5.

**Fig 6:**
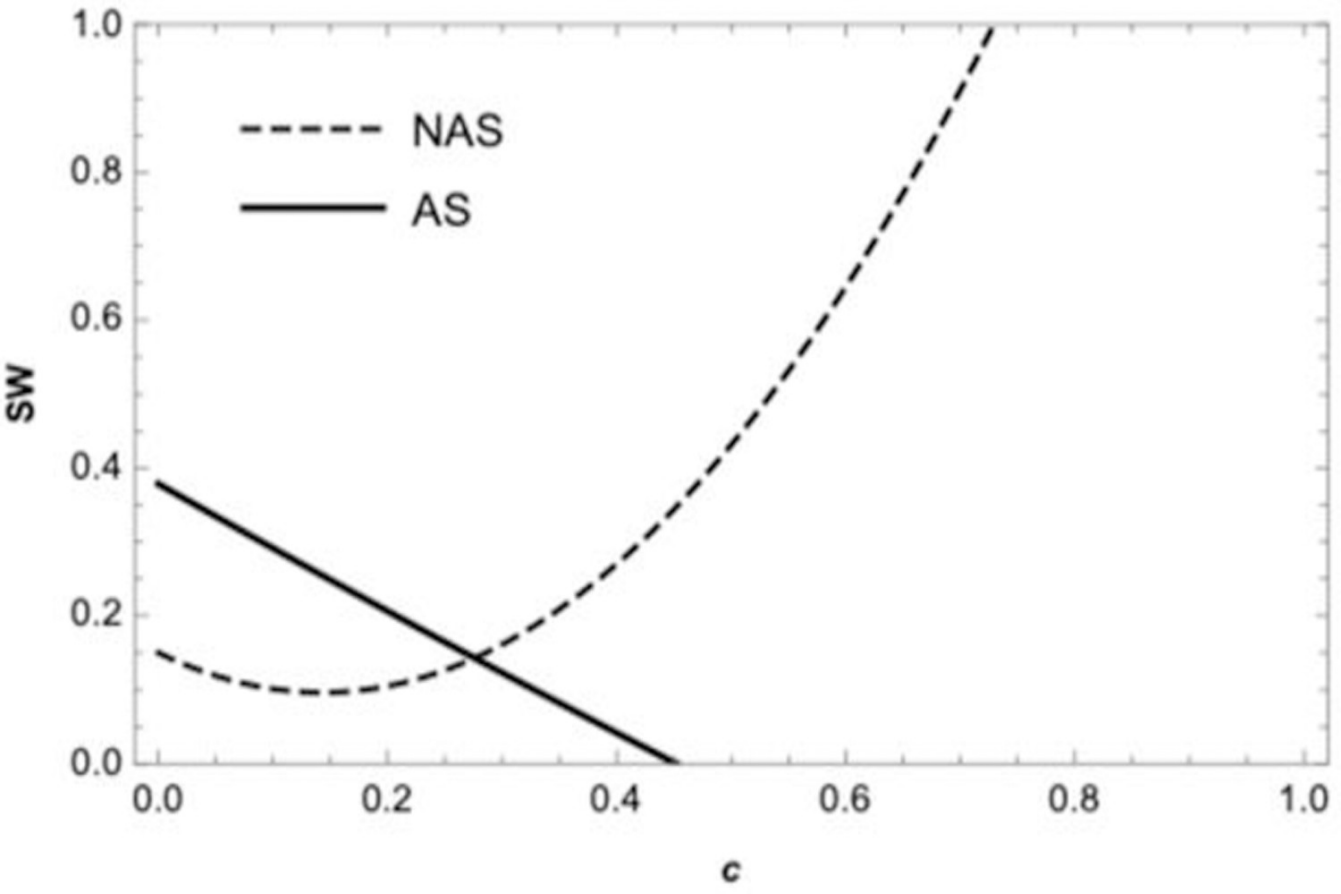
The social welfare in decentralized supply chain.

**Fig 7:**
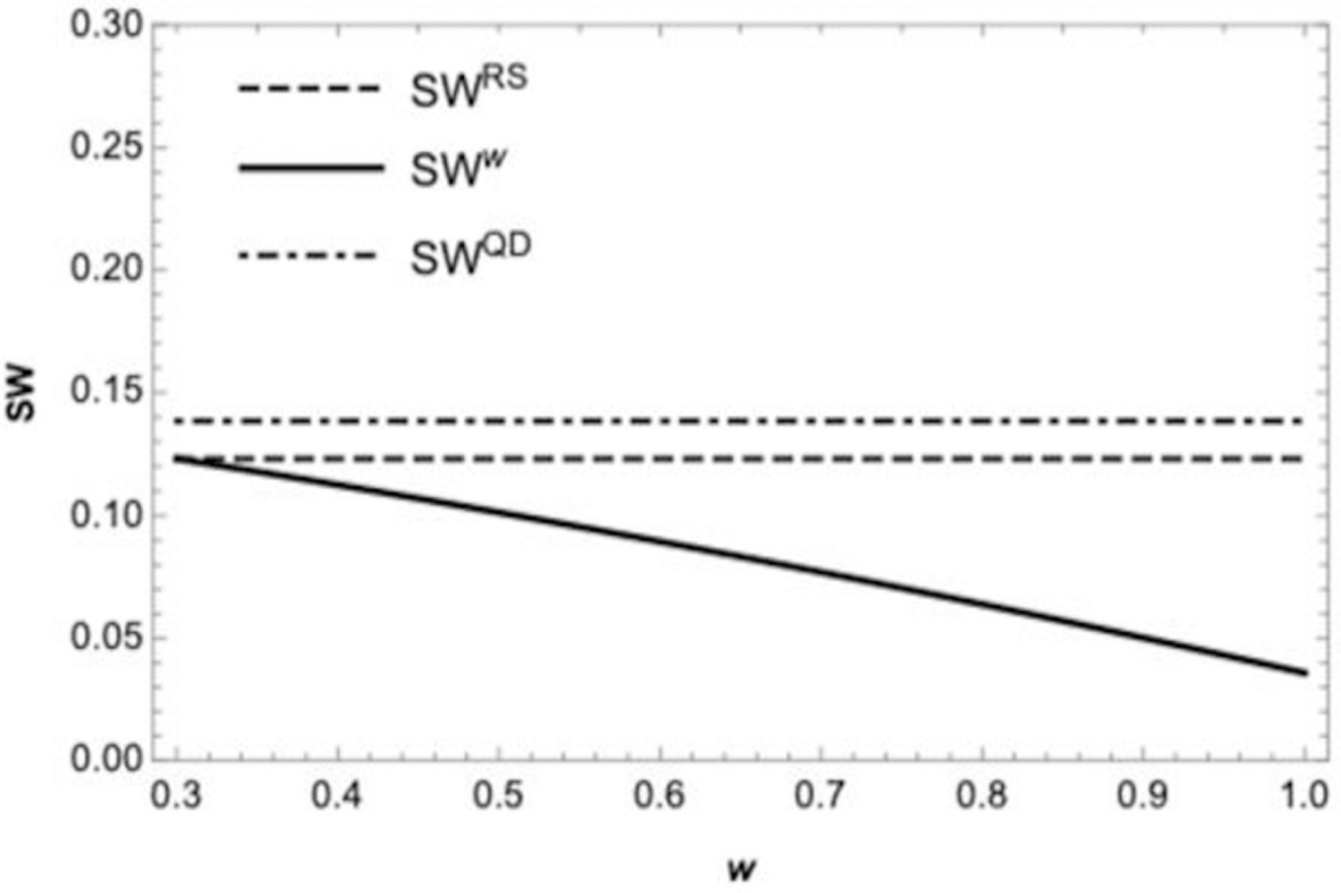
The social welfare under different contracts if the retailer sells in advance.

Fig 6 depicts the social welfare under the centralized supply chain. The solid curve represents the social welfare of the centralized supply chain if the retailer sells in advance, and the dashed represents the social welfare if the retailer does not sell in advance, which corresponds to the cases in proposition 6. As shown in Fig 7, The solid curve represents the social welfare of the wholesale price contract if the retailer sells in advance, the dashed represents the social welfare of the quantity discount contract, and the dot-dashed corresponding to the social welfare of the revenue-sharing contract, the social welfare of revenue-sharing contracts is greater than wholesale price contracts but less than quantity discount contracts, which is consistent with the conclusion of proposition 7.

## 7. Discussion and conclusion

The AS has been widely applied in fresh industry for it can elevating the customer experience and increase flexibility thus profit for a retailer. However, intense price competition not only disturbs the normal market price order, but also causes many adverse effects on suppliers. Thus, effective channel management and contracts plays an important role in controlling AS market activities. Designing an appropriate choice of contracts is crucial for suppliers that distribute FAP when the retailer sells in advance.

In this paper, we investigate the boundary condition for retailers to sell in advance under three classic contracts, discuss how the supplier designs contract when take the AS into consideration. We find that the AS has a significant impact on classic contracts. Revenue sharing contracts can coordinate the supply chain and are superior to wholesale price contracts and quantity discount contracts. By setting appropriate parameters, a quantity discount contract can coordinate the supply chain if the retailer does not sell in advance. But such a quantity discount contract fails to coordinate the supply chain if the retailer sells in advance and makes the retailer may be more inclined to use the wholesale price contract, despite the double marginal effect of the wholesale price contract. Besides, the effects of AS on supply chain’s profit depends on the difference of contract. In the wholesale price contract, when the procurement cost is low and the freshness loss of FAP is small in spot market, the supply chain is harmed by the AS. Under the quantity discount contract, the supplier is always worse off with AS, the effects of the AS on retailer’s profit are uncertain and depends on whether they benefit from the AS outweighs the profit loss to spot market, therefore, the impact of the AS on the profit of the total supply chain is uncertain. Since the revenue sharing contract can perfectly coordinate the supply chain whenever the retailer decides to sell in advance or not, the AS does not affect the supply chain. Furthermore, our analysis suggests that although the revenue sharing contract can perfectly coordinate the supply chain when retailer sells in advance, it does not always result in highest social welfare of the three contracts.

Our model is based on the research that all consumers are strategic, which will pay attention to the pre-sale information and decides whether to purchase during the pre-sale period. Our analysis can be extended to the setting that one segment of the consumers is strategic, and one is short-sighted. Short-sighted consumers do not care about the pre-sale information and only buy in the sales period. Therefore, analyzing the impact of AS markets on a supply chain when consumers’ type is different can be explored in future research. Besides, we can extend our model to the supply chain structures of competing retailers to study price competition on a supply chain when the retailer sells in advance. In practice, many large e-commerce retailers of FAP such as Taobao, Jindong and Meituan have established cooperative relationships with WeChat, Weibo and other social platforms for AS, and retailers pay the commission to the social platform. Thus, future research can also consider social platforms and analyze AS strategies with the commission rates and network externalities of social platforms.

## Supporting information

S1 Data (DOCX)

S1 Appendix (DOCX)
