# Supplementary material for: Contract choice and advance selling strategy in a supply chain of FAP
Source: PLoS One. 2022 Mar 24;17(3):e0265661. doi: 10.1371/journal.pone.0265661 (PMC8947360; doi:10.1371/journal.pone.0265661)
Supplement: S1 Data — (DOCX) [file pone.0265661.s001.docx]

Data set

We built a two-stage game theory model to analyze the impacts of AS on three classic contracts, examines the boundary conditions of a retailer should sell in advance or not, and some useful results are obtained. Readers can replicate the results of our study through the proofs in the appendix section.

In numerical analysis of this paper, the original data of parameters are:$a=0.1$, $c=0.3$, $b=0.2$. Substituting these data to equations we obtained in the paper, the readers can redraw the figures in this paper.
