# Supplementary material for: Contract choice and advance selling strategy in a supply chain of FAP
Source: PLoS One. 2022 Mar 24;17(3):e0265661. doi: 10.1371/journal.pone.0265661 (PMC8947360; doi:10.1371/journal.pone.0265661)
Supplement: S1 Appendix — (DOCX) [file pone.0265661.s002.docx]

**Appendix**

**Proof of proposition 1**

Taking the AS into consideration, in period 2, the supply chain determines the spot price $p_{r}^{c}$ to maximize profit, the profit of spot market is:

|  | $\pi_{SC2}^{c}=p_{r}^{c}D_{r}=p_{r}^{c}(\frac{p_{c}^{c}-p_{r}^{c}}{1-a}-\frac{p_{r}^{c}}{a})$ | (A.1) |
| --- | --- | --- |

Since $\frac{\partial^{2}\pi_{SC2}^{c}}{\partial^{2}p_{r}^{c}}<0$, the optimal spot price satisfies the first-order condition is:

|  | $p_{r}^{c}=\frac{1}{2}ap_{c}^{c}$ | (A.2) |
| --- | --- | --- |

In period 1, the centralized supply chain maximizes its profit by deciding $p_{c}^{c}$:

|  | $\pi_{SC1}^{c}=p_{c}^{c}D_{c}-c(D_{c}+D_{r})+\pi_{SC2}^{c}$ | (A.3) |
| --- | --- | --- |

Substituting (A.2) into (A.3), we obtain $\pi_{SC1}^{c}=\frac{1}{4}[2p_{c}^{c}(2+c)-4c-\frac{\left( 4-3a \right){p_{c}^{c}}^{2}}{1-a}]$.

Since $\frac{\partial^{2}\pi_{SC}^{c}}{\partial^{2}p_{c}^{c}}=-\frac{4-3a}{2(1-a)}<0$, the optimal pre-order price satisfies the first-order condition is:

|  | $p_{c}^{c}=\frac{(1-a)(2+c)}{4-3a}$ | (A.4) |
| --- | --- | --- |

According to equations (A.2), (A.3), (A.4), the optimal spot price andthe optimal centralized supply chain’s profit is:

|  | $p_{r}^{c}=\frac{a(1-a)(2+c)}{2(4-3a)}$, $\pi_{SC}^{c}=\frac{4-12c+c^{2}-a(4-8c+c^{2})}{4(4-3a)}$ | (A.5) |
| --- | --- | --- |

Comparing equilibrium results between the centralized supply chain with AS and without AS, we have $\pi_{SC}^{c}-\pi_{SC}^{c^{N}}=-\frac{\left( 2-a \right)[3a^{2}+2c^{2}-a(2-2c+c^{2})]}{4a(4-3a)}$, if $0<a<\frac{2}{3}$ and $c<\frac{\sqrt{a(4-7a+3a^{2})}-a}{2-a}=c_{1}$,$\pi_{SC}^{c}-\pi_{SC}^{c^{N}}>0$. 🞎

**Proof of proposition 2**

The supplier charges the retailer a wholesale price $w$. In period 2, the retailer determines his optimal price $p_{r}^{w}$:

|  | $\pi_{r2}^{w}=p_{r}^{w}D_{r}=p_{r}^{w}(\frac{p_{c}^{w}-p_{r}^{w}}{1-a}-\frac{p_{r}^{w}}{a})$ | (A.6) |
| --- | --- | --- |

Since $\frac{\partial^{2}\pi_{r2}^{w}}{\partial^{2}p_{r}^{w}}<0$, the optimal spot price satisfies the first-order condition is:

|  | $p_{r}^{w}=\frac{1}{2}ap_{c}^{w}$ | (A.7) |
| --- | --- | --- |

In period 1, the retailer maximizes his profit by deciding $p_{c}^{w}$:

|  | $\pi_{r1}^{w}=p_{c}^{w}D_{c}+\pi_{r2}^{w}-w(D_{c}+D_{r})$ | (A.8) |
| --- | --- | --- |

Substituting (A.7) into (A.8), we obtain $\frac{\partial^{2}\pi_{r1}^{w}}{\partial^{2}p_{c}^{w}}<0$, thus the optimal pre-order price satisfies the first-order condition is:

|  | $p_{c}^{w}=\frac{(1-a)(2+w)}{4-3a}$ | (A.9) |
| --- | --- | --- |

The supplier’s profit function is:

|  | $\pi_{s}^{w}=(w-c)\left( D_{c}+D_{r} \right)=(w-c)(1-\frac{p_{r}^{w}}{a})$ | (A.10) |
| --- | --- | --- |

Substituting (A.9) into (A.7), we obtain $p_{r}^{w}=\frac{a(1-a)(2+w)}{2(4-3a)}$. If $w=c$ then $p_{r}^{w}=p_{r}^{c}$ and the wholesale price contract fails to coordinate the supply chain.

Substituting the optimal $p_{r}^{w}$ and $p_{c}^{w}$ into (A.8) and (A.10), we can get:

|  | $\pi_{r}^{w}=\frac{4-12w+w^{2}-a\left( 4-8w+w^{2} \right)}{16-12a}$,$\pi_{s}^{w}=\frac{\left( w-c \right)[6-w-a\left( 4-w \right)]}{2(4-3a)}$ | (A.11) |
| --- | --- | --- |
|  | $\pi_{sc}^{w}=\frac{4-2c(6-w)-w^{2}-a[4-2c(4-w)-w^{2}]}{4(4-3a)}$ |  |

Comparing equilibrium results between the supply chain with AS and without AS in wholesale price contract, we have $\pi_{r}^{w}-\pi_{r}^{w^{N}}=\frac{\left( a-2 \right)[3a^{2}+2w^{2}-a\left( 2-2w+w^{2} \right)]}{4a(4-3a)}$. if $0<a<\frac{2}{3}$ and $w<\frac{\sqrt{4a-7a^{2}+3a^{3}}-a}{2-a}=\tilde{w}$,,$\pi_{r}^{w}-\pi_{r}^{w^{N}}>0$. 🞎

**Proof of corollary 1**

Since $p_{r}^{w}-p_{r}^{w^{N}}$ increases with $w$, when $w=1$, $p_{r}^{w}-p_{r}^{w^{N}}=\frac{3a^{2}-3a\left( 2-c \right)-4c}{4\left( 4-3a \right)}<0$, thus $p_{r}^{w}<p_{r}^{w^{N}}$ for $w\in(0,1)$.

| $\pi_{SC}^{w}-\pi_{SC}^{w^{N}}=-\frac{(2-a)\left( 3a^{2}+2(2c-w)w+a[2c(1-w)+w^{2}-2] \right)}{4a(4-3a)}$ | (A.12) |
| --- | --- |

Since $\frac{\partial\pi_{SC}^{w}-\pi_{SC}^{w^{N}}}{\partial c}=-\frac{\left( 2-a \right)[4w+2a(1-w)]}{4a(4-3a)}<0$, which means $\pi_{SC}^{w}-\pi_{SC}^{w^{N}}$ monotonically decreases with $c$. $\pi_{SC}^{w}-\pi_{SC}^{w^{N}}=0$ has one roots $c_{2}=\frac{2w^{2}-3a^{2}+a(2-w^{2})}{4w+2a(1-w)}>0$ if $a<\frac{1}{6}(2-w^{2}+\sqrt{4+20w^{2}+w^{4}})$. Therefore, if $a<\frac{1}{6}(2-w^{2}+\sqrt{4+20w^{2}+w^{4}})$ and $c_{2}<c<1$, $\pi_{SC}^{w}-\pi_{SC}^{w^{N}}<0$. 🞎

**Proof of corollary 2**

$\frac{dp_{c}^{w}}{da}=-\frac{2+w}{{(4-3a)}^{2}}<0$,$\frac{d\pi_{r}^{w}}{da}=-\frac{{(2+w)}^{2}}{4{(4-3a)}^{2}}<0$ ,$\frac{d\pi_{s}^{w}}{da}=\frac{(w-c)(2+w)}{2{(4-3a)}^{2}}>0$. 🞎

**Proof of proposition 3**

Under the quantity discount contract, in period 2, the retailer determines his spot price $p_{r}^{QD}$ to maximize its profit, the profit of spot market is:

|  | $\pi_{r2}^{QD}=p_{r}^{QD}D_{r}=p_{r}^{QD}(\frac{p_{c}^{QD}-p_{r}^{QD}}{1-a}-\frac{p_{r}^{QD}}{a})$ | (A.13) |
| --- | --- | --- |

Since $\frac{\partial^{2}\pi_{r2}^{QD}}{\partial^{2}p_{r}^{QD}}<0$, the optimal spot price satisfies the first-order condition is:

|  | $p_{r}^{QD}=\frac{1}{2}ap_{c}^{QD}$ | (A.14) |
| --- | --- | --- |

In period 1, the centralized supply chain maximizes profit by deciding $p_{c}^{c}$:

|  | $\pi_{r1}^{QD}=p_{c}^{QD}D_{c}+\pi_{r2}^{QD}-w(q_{r})(D_{c}+D_{r})$ | (A.15) |
| --- | --- | --- |

Substituting (A.14) into (A.15), we obtain:

| $\pi_{r1}^{QD}=\frac{1}{4}\left[ 2c(1-b)(p_{c}^{QD}-2)+\frac{p_{c}^{QD}\left( 4-2b-a[4-3p_{c}^{QD}-b\left( 2-p_{c}^{QD} \right)]+(b-4)p_{c}^{QD} \right)}{1-a} \right]$ | (A.16) |
| --- | --- |

Since $\frac{\partial^{2}\pi_{r1}^{QD}}{\partial^{2}p_{c}^{QD}}=-\frac{4-a(3-b)-b}{2(1-a)}<0$, the optimal pre-order price satisfies the first-order condition is:

|  | $p_{c}^{QD}=\frac{(1-a)(2-b+c-bc)}{4-a(3-b)-b}$ | (A.17) |
| --- | --- | --- |

Substituting (A.17) into (A.15), we obtain $p_{r}^{QD}=\frac{a\left( 1-a \right)[2+c-b(1-c)]}{2[4-b-a(3-b)]}$, if $w(q_{r})=c$, then $p_{r}^{QD}=p_{r}^{c}$ and the quantity discount contract can coordinate the supply chain.

The supplier’s profit function is:

|  | $\pi_{s}^{QD}=\left[ w\left( q_{r} \right)-c \right]\left( D_{c}+D_{r} \right)=[b(1-c)+c-bq_{r}-c](1-\frac{p_{r}^{QD}}{a})$ | (A.18) |
| --- | --- | --- |

Substituting the optimal $p_{r}^{QD}$ and $p_{c}^{QD}$ into (A.16) and (A.18), we can get:

| $\pi_{r}^{QD}=\frac{4+b^{2}{(1-c)}^{2}+c(c-12)-2b(2-7c+c^{2})-a[4+b^{2}{(1-c)}^{2}+c(c-8)-2b(2-5c+c^{2})]}{4[4-a(3-b)-b]}$ | (A.19) |
| --- | --- |
| $\pi_{s}^{QD}=\frac{b\left[ b-6+a\left[ 4-b\left( 1-c \right)-c \right]+c(1-b) \right][b-2+a[2-b(1-c)-5c]+c(7-b)]}{4{[4-a(3-b)-b]}^{2}}$ |  |
| $\pi_{SC}^{DQ}=\frac{\left( \begin{aligned} a^{2}\left[ \begin{aligned} (3-2b)(4-8c+c^{2}) \\ +b^{2}(1-4c-c^{2}) \end{aligned} \right]-2[b^{2}c\left( 3+c \right)+(b-2)(4-12c+c^{2})] \\ +a[68c-28-7c^{2}+4b(4-10c+c^{2})+b^{2}(10c+3c^{2}-1)] \end{aligned} \right)}{4{[4-a(3-b)-b]}^{2}}$  $\pi_{r}^{QD}-\pi_{r}^{QD^{N}}=-\frac{\left( \begin{aligned} ac\left[ 4c-b^{2}(2+c)+2b(4+c) \right]+a^{3}\left[ 8-2c-b^{2}(2-c)c+c^{2}-2b(1-4c+c^{2}) \right] \\ -a^{4}\left( 3-b \right)-(4-b)bc^{2}-a^{2}[4-2c\left( 2-9b+2b^{2})+\left( 4-3b+b^{2} \right)c^{2} \right)] \end{aligned} \right)}{4a^{2}\left[ 4-a(3-b)-b \right]}$ |  |

Define $B=\left( \begin{aligned} ac\left[ 4c-b^{2}(2+c)+2b(4+c) \right]+a^{3}\left[ 8-2c-b^{2}(2-c)c+c^{2}-2b(1-4c+c^{2}) \right] \\ -a^{4}\left( 3-b \right)-(4-b)bc^{2}-a^{2}[4-2c\left( 2-9b+2b^{2})+\left( 4-3b+b^{2} \right)c^{2} \right)] \end{aligned} \right)$, since $\frac{\partial^{2}B}{\partial b^{2}}=2{(1-a)}^{2}c[c-a(2-c)]$, thus if $0<c<\frac{2a}{1+a}$, then $\frac{\partial^{2}B}{\partial b^{2}}<0$; if $\frac{2a}{1+a}<c<1$, $\frac{\partial^{2}B}{\partial b^{2}}>0$. Note that $B$ is a quadratic equation of $b$, which has two roots $b_{1}>1$ if $\frac{2a}{1+a}<c<1$ and $b_{1}<0$ if $0<c<\frac{2a}{1+a}$, $b_{2}<1$. We have $B>0$ for $b\in(0,b_{2})$, $B<0$ for $b\in(b_{2},1)$, and $\pi_{r}^{QD}-\pi_{r}^{QD^{N}}<0$ for $b\in(0,b_{2})$, $\pi_{r}^{QD}-\pi_{r}^{QD^{N}}>0$ for $b\in(b_{2},1)$. 🞎

**Proof of corollary 3**

Since $p_{r}^{QD}-p_{r}^{c}=\frac{b\left( 1-a \right)[a-2-c(3-2a)]}{\left( 4-3a \right)[4-a(3-b)-b]}<0$, thus $p_{r}^{QD}<p_{r}^{c}$.

Note that $p_{r}^{QD}-p_{r}^{QD^{N}}=\frac{\left( 1-a \right)^{2}bc-\left( 2-a \right)\left[ \left( 2-a \right)c+a \right]}{8-2a\left( 3-b \right)-2b}<0$, thus $p_{r}^{QD}<p_{r}^{QD^{N}}$.

For $\pi_{s}^{QD}-\pi_{s}^{QD^{N}}=0$ is a quadratic equation of $c$, which have no real roots for $c\in(0,1)$. Since $\frac{\partial^{2}\pi_{s}^{QD}-\pi_{s}^{QD^{N}}}{\partial c^{2}}<0$, thus $\pi_{s}^{QD}<\pi_{s}^{QD^{N}}$. 🞎

**Proof of proposition 4**

Under the revenue-sharing contract, in period 2, the retailer determines his spot price $p_{r}^{RS}$ to maximize its profit, the profit of spot market is:

|  | $\pi_{r2}^{RS}=p_{r}^{RS}D_{r}=p_{r}^{RS}(\frac{p_{c}^{RS}-p_{r}^{RS}}{1-a}-\frac{p_{r}^{RS}}{a})$ | (A.20) |
| --- | --- | --- |

Since $\frac{\partial^{2}\pi_{r2}^{RS}}{\partial^{2}p_{r}^{RS}}<0$, the optimal spot price satisfies the first-order condition is:

|  | $p_{r}^{RS}=\frac{1}{2}ap_{c}^{RS}$ | (A.21) |
| --- | --- | --- |

In period 1, the retailer maximizes its profit by deciding $p_{c}^{RS}$:

|  | $\pi_{r1}^{RS}=\lambda p_{c}^{RS}D_{c}-w^{RS}\left( D_{c}+D_{r} \right)+\lambda\pi_{r2}^{RS}$ | (A.22) |
| --- | --- | --- |

Substituting (A.21) into (A.22), we obtain $\pi_{r1}^{RS}=\frac{1}{4}[\frac{\lambda p_{c}^{RS}[4-4a+(3a{-4)p}_{c}^{RS}]}{1-a}-2w(2-p_{c}^{RS})]$, Since $\frac{\partial^{2}\pi_{r1}^{RS}}{\partial^{2}p_{c}^{RS}}=-\frac{(4-3a)\lambda}{2(1-a)}<0$, the optimal pre-order price satisfies the first-order condition is:

|  | $p_{c}^{RS}=\frac{(1-a)(w+2\lambda)}{(4-3a)\lambda}$ | (A.23) |
| --- | --- | --- |

Substituting (A.23) into (A.21), we obtain $p_{r}^{RS}=\frac{a(1-a)(w+2\lambda)}{2(4-3a)\lambda}$. if $w^{RS}=c\lambda$, then $p_{r}^{RS}=p_{r}^{c}$ and the revenue-sharing contract can coordinate the supply chain.

The supplier’s profit function is:

|  | $\pi_{s}^{RS}=\left( w^{RS}-c \right)\left( D_{c}+D_{r} \right)+(1-\lambda)(p_{c}^{RS}D_{c}+p_{r}^{RS}D_{r})$ | (A.24) |
| --- | --- | --- |

Substituting the optimal $p_{r}^{RS}$ and $p_{c}^{RS}$ into (A.22) and (A.24), the optimal profit of supplier, retailer and the supply chain is:

| $\pi_{r}^{RS}=\lambda\pi_{SC}^{c}$, $\pi_{s}^{RS}=(1-\lambda)\pi_{SC}^{c}$, $\pi_{SC}^{RS}=\pi_{SC}^{c}$ | (A.25) |
| --- | --- |

🞎

**Proof of proposition 5**

$\pi_{SC}^{w}-\pi_{SC}^{QD}=0$ is a quadratic equation of $w$, which has two roots $w_{1}=\frac{c(4-3a)-b[2-a+c(4-3a)]}{4-a(3-b)-b}$, $w_{2}=\frac{c(4-3a)+b(2-a)(1+c)}{4-a(3-b)-b}$. Note that $w_{1}<c<w_{2}$, since $\frac{\partial^{2}\pi_{SC}^{w}-\pi_{SC}^{QD}}{\partial w^{2}}=-\frac{2(1-a)}{4(4-3a)}<0$, there must exist a point $w\in(c,w_{2})$ such that $\pi_{SC}^{w}>\pi_{SC}^{QD}$. 🞎

**Proof of proposition 6**

Comparing consumer welfare and social welfare between the centralized supply chain with AS and without AS, we have $CS^{C^{N}}-CS^{C}=-\frac{(4-3c)c+a[3-2c(1-c)]}{8(4-3a)}<0$, $SW^{C^{N}}-SW^{C}=\frac{a^{2}\left( 13-2c \right)+8c^{2}+a\left( 4c-5c^{2}-8 \right)-6a^{3}}{8a\left( 4-3a \right)}$. Note that $SW^{C^{N}}-SW^{C}=0$ is a quadratic equation of $c$, which has two roots $0<c_{4}=\frac{a(a-2)+\sqrt{64a-140a^{2}+109a^{3}-29a^{4}}}{8-5a}<1$, $c_{5}=\frac{a\left( a-2 \right)-\sqrt{64a-140a^{2}+109a^{3}-29a^{4}}}{8-5a}<0$. Since $\frac{\partial^{2}SW^{C^{N}}-SW^{C}}{\partial c^{2}}>0$, there must exist a critical crossing point $c_{4}$ such that $SW^{C^{N}}>SW^{C}$ for $c\in(c_{4},1)$, and $SW^{C^{N}}<SW^{C}$ for $c\in(0,c_{4})$. 🞎

**Proof of proposition 7**

Comparing social welfare between the wholesale price contract and the revenue sharing contract when retailer sells in advance, we have $SW^{RS}-SW^{w}=\frac{\left( 1-a \right)[3c^{2}-4c(1+w)+w(4+w)]}{8(4-3a)}$. Note that $SW^{RS}-SW^{w}=0$ is a quadratic equation of $w$, which has two roots $w_{3}=c$, $w_{4}=3c-4$. Since $\frac{\partial^{2}SW^{RS}-SW^{w}}{\partial w^{2}}=\frac{2(1-a)}{8(4-3a)}>0$, there must exist a critical crossing point$,$ such that $SW^{RS}>SW^{w}$ for $w>c$. Comparing social welfare between the revenue sharing contract and the quantity discount contract when retailer sells in advance, we have:

| $SW^{RS}-SW^{QD}=\frac{b\left( 1-a \right)\left( a-2-3c+2ac \right)[5ab+(8-6a)(2-c)-b(6+c)]}{8(4-3a){[4-a(3-b)-b]}^{2}}$ | (A.26) |
| --- | --- |

Since $SW^{RS}-SW^{QD}=0$ is a quadratic equation of $c$, which has two roots $c_{6}=-\frac{2-a}{3-2a}<0$, $c_{7}=2-\frac{b(8-5a)}{8-6a+b}>1$. Since $\frac{\partial^{2}SW^{RS}-SW^{QD}}{\partial c^{2}}=\frac{b\left( 1-a \right)[24+12a^{2}+3b-2a(17+b)]}{4(4-3a){[4-a(3-b)-b]}^{2}}>0$, there have $SW^{RS}<SW^{QD}$. 🞎
